# Non-alcoholic fatty liver disease in mice with heterozygous mutation in TMED2

**DOI:** 10.1371/journal.pone.0182995

**Published:** 2017-08-10

**Authors:** Wenyang Hou, Swati Gupta, Marie-Claude Beauchamp, Libin Yuan, Loydie A. Jerome-Majewska

**Affiliations:** 1 Department of Human Genetics, McGill University, Montreal, Québec, Canada; 2 Department of Pediatrics, McGill University Health Centre Glen Site, Montreal, Québec, Canada; 3 Department of Anatomy and Cell Biology, McGill University, Strathcona Anatomy and Dentistry Building, Québec, Canada; Wayne State University, UNITED STATES

## Abstract

The transmembrane emp24 domain/p24 (TMED) family are essential components of the vesicular transport machinery. Members of the TMED family serve as cargo receptors implicated in selection and packaging of endoplasmic reticulum (ER) luminal proteins into coatomer (COP) II coated vesicles for anterograde transport to the Golgi. Deletion or mutations of Tmed genes in yeast and Drosophila results in ER-stress and activation of the unfolded protein response (UPR). The UPR leads to expression of genes and proteins important for expanding the folding capacity of the ER, degrading misfolded proteins, and reducing the load of new proteins entering the ER. The UPR is activated in non-alcoholic fatty liver disease (NAFLD) in human and mouse and may contribute to the development and the progression of NAFLD. *Tmed2*, the sole member of the vertebrate Tmed β subfamily, exhibits tissue and temporal specific patterns of expression in embryos and developing placenta but is ubiquitously expressed in all adult organs. We previously identified a single point mutation, the 99J mutation, in the signal sequence of *Tmed2* in an N-ethyl-N-nitrosourea (ENU) mutagenesis screen. Histological and molecular analysis of livers from heterozygous mice carrying the 99J mutation, *Tmed2*^*99J/+*^, revealed a requirement for TMED2 in liver health. We show that *Tmed2*^*99J/+*^ mice had decreased levels of TMED2 and TMED10, dilated endoplasmic reticulum membrane, and increased phosphorylation of eIF2α, indicating ER-stress and activation of the UPR. Increased expression of *Srebp1a* and *2* at the newborn stage and increased incidence of NAFLD were also found in *Tmed2*^*99J/+*^ mice. Our data establishes *Tmed2*^*99J/+*^ mice as a novel mouse model for NAFLD and supports a role for TMED2 in liver health.

## Introduction

The ten TMED proteins in mouse and human are subdivided into four subfamilies based on sequence similarity [1]: three belong to the α subfamily (TMED4, 9, 11); one to the β family (TMED2); five to the γ subfamily (TMED1, 3, 5, 6, 7); and one to the δ family (TMED10). TMED proteins were found to form monomers, dimers and heterodimers [2]and to regulate the stability of each other [3]. Thus, loss of one member of a subfamily resulted in loss of TMED proteins in other subfamilies [2, 4, 5].

Members of the TMED family serve as cargo receptors implicated in selection and packaging of endoplasmic reticulum (ER) luminal proteins into COP II coated vesicles for anterograde transport to the Golgi. TMED putative cargos include WNTs and glycosylphosphatidylinositol-anchored proteins (GPI-AP)[3]. Deletion or mutations of Tmed genes in yeast and Drosophila resulted in ER-stress and activation of the unfolded protein response (UPR)[6, 7].The UPR leads to expression of genes and proteins important for expanding the folding capacity of the ER, degrading misfolded proteins, and reducing the load of new proteins entering the ER [8].

*Tmed2*, the sole member of the vertebrate Tmedβ subfamily exhibits tissue and temporal specific patterns of expression in embryos and developing placenta [5, 9, 10] but was ubiquitously expressed in all adult organs [11]. Our group identified a point mutation in the signal sequence of *Tmed2* in a mutant mouse line, 99J, generated in a mutagenesis screen with N-ethyl-N-nitrosourea (ENU). We showed that the 99J mutation results in decreased TMED2 protein levels in heterozygous (*Tmed2*^*99J/+*^) embryos and loss of TMED2 protein in homozygous mutant embryos (*Tmed2*^*99J/99J*^)[5]. *Tmed2* is required for morphogenesis of the embryo and its associated placenta, and consequently *Tmed2*^*99J/99J*^ embryos arrest at mid-gestation, shortly after embryonic day (E)10.5 [5]. Similarly, *Tmed10*, the sole member of the Tmed δ family is required for embryonic development and, *Tmed10* homozygous mutant embryos arrest early in development, before E3.5 [4]. Furthermore, *Tmed10* heterozygous mice showed dilated Golgi and reduced amount of at least two other members of the TMED family, TMED9 and TMED3. The consequences of reduced levels of TMED2 have not been described yet. Herein, we report that in adult mice, normal amount of TMED2 was required for liver health. Mice heterozygous for the ENU-induced *Tmed2*^*99J/+*^ allele had decreased levels of TMED2 and TMED10, dilated endoplasmic reticulum membrane and increased phosphorylation of eIF2α (indicating ER-stress and activation of the UPR), increased expression of *Srebp1a* and *2* at the newborn stage, and an increased incidence of non-alcoholic fatty liver disease (NAFLD).

NAFLD is the major cause of chronic liver disease worldwide in both developing and developed countries [12, 13]. Although, the global prevalance of NAFLD is estimated to be 25.24%, with the highest prevalences in South America (30.45%) and the Midle East (31.79%) [14], no therapy exists to treat NAFLD. Lifestyle changes, including diet and exercise, can result in significant improvement in steatosis in a subset of patients and is the treatment currently recommend for NAFLD [15]. In addition, two drugs Pioglizatone and Obeticholic Acid, were also found to significantly improve histological signs of NAFLD in two separate randomized, placebo-controled trials [16, 17]. The thiazolidinedione, Pioglitazone, was shown to be safe and effective in patients with prediabetes or type 2 diabetes and non-alcoholic steatohepatitis [16], howevere, concerns persist regarding the longterm safety of this drug. In addition, the Farnesoid X nuclear receptor ligand Obeticholic Aid, also resulted in signficant improvement of histological features of NAFLD [17]. Nonetheless, since many patients are refractory to these treatments and NAFLD is a heterogenous disease, identifying and characterizing novel models for NAFLD will aid in development of biomarkers and new therapeutic targets. Our work indicates that *Tmed2* heterozygous mice with the 99J mutation is a novel mouse model for NAFLD and supports a role for TMED2 in liver health.

## Materials and methods

### Mice

All procedures and experiments were performed according to the guidelines of the Canadian Council on Animal Care and approved by the Animal Care Committee of the Montreal Children’s Hospital. The 99J mouse line was generated on a C57/BL6J genetic background and maintained on a mixed C3H genetic background (C3HeB/FeJ and C3HeB/FeV). The 99J mutation was genotyped by PCR using primers to D5MIT95 and D5MIT213 as previously described [5]. For newborns, the date of birth was designated as P1. Liver analysis was performed on samples collected from mice between 1–17 months of age euthanized between 13h - 17h on the day of tissue collection. For molecular analysis, livers collected at P5 were classified as pre-weaning newborn, livers collected from animals between 1 and 2 months of age were classified as post-weaning juveniles, and livers collected from animals 3–6 months of age were classified as mature adults.

### Cell lines

Human hepatocellular carcinoma cells (HepG2) and the liver adenocarcinoma cells (SK-HEP-1) (ATCC, Manassas, VA, USA) were used in this study (Gifts of Dr. P. Metrakos). Both cell lines were grown in Minimal Essential Medium supplemented with 10% of FBS, 1% penicillin/streptomycin (Wisent, Saint-Bruno, Quebec, Canada). Each cell line was passaged every 4 to 6 days. Cells were maintained at 37°C in a 5% CO_2_, 95% air atmosphere incubator. Treatment with tunicamycin (Sigma, Oakville, Ontario, Canada), diluted in DMSO, was performed in cell medium at the indicated concentrations. DMSO was used as vehicle.

### Tunicamycin

Tunicamycin was prepared in 150mM sucrose and was injected intraperitoneally at a dose of 0.75mg/kg in 10 weeks old wildtype (n = 4) and *Tmed2*^*99J/+*^ (n = 6) mice at day 0. The weight of mice was monitored daily in the morning for 14 days at which point they were euthanized and organs collected.

### Liver collection

Liver samples collected from individual adult animals were used for multiple experiments. To standardize experiments between animals, the left lateral lobe of the liver was used for histology, the left medial lobe was used for RNA analysis, and the rest of the liver tissue was used for Western blot analysis. Liver tissue was collected and fixed in 4% PFA or Bouin solution (Ricca Chemical, Texas, USA) for immuno-histochemical analyses and/or histology, respectively. For Western blot analysis, liver was flash frozen in liquid nitrogen before lysis in RIPA buffer. For RNA analysis, liver tissue was treated with Trizol and stored at -80°C before RNA extraction. For TEM liver samples, mice were anesthetized and the internal organs were perfused and fixed with 25% glutaraldehyde in 0.1 M cacodylate buffer.

### Paraffin and cryoembedding

Liver samples fixed in Bouin and/or PFA were dehydrated and embedded in paraffin, as previously described [5, 18, 19]. All samples were sectioned at 5μm thickness. For cryosection, liver samples were fixed in 4% PFA, cryoprotected in 30% sucrose, and cryoembedded in plastic molds before sectioning at 10μm thickness.

### Transmission electron microscopy (TEM)

Livers collected from wild type (n = 2) and *Tmed2*^*99J/+*^(n = 4) mice were washed in Phosphate Buffered Saline (PBS), fixed with 25% glutaraldehyde in 0.1 M cacodylate buffer (pH = 7.5), stained in 2% reduced osmium tetraoxide, and embedded in Epone. The samples were sectioned at the McGill FEMR facility. Imaging of sections was completed on the Tecnai T12 120 kV TEM microscope.

### Scoring for NAFLD

Paraffin embedded samples were stained with Hematoxylin and Eosin (H&E) using standard protocols and scored for NAFLD using a scoring system adapted from Kleiner et al., 2005, for details see Table 1 [20]. Liver samples were scored using 20X objectives on a Zeiss Axiophot compound microscope. Minimum of two slides (four sections each) at least 100 μm apart were used for scoring liver samples. Representative images were taken for each sample using a Zeiss Axiophot compound microscope, AxioCamMRc camera and Axiovision v4.7.1.0 software. Samples with scores of ≥4 were considered sick and samples with scores of < 4 were diagnosed healthy. All livers were scored by two individuals blind to animal genotype.

**Table 1:** Scoring system used for phenotypic analysis of *Tmed2*^*99J/+*^ and wildtype livers.

Histological Features		Score	
Steatosis	Macrovesicular	<5% of cells	0
		5%-33% of cells	1
		33%-66% of cells	2
		>66% of cells	3
	Microvesicular	<5% of cells	0
		5%-33% of cells	1
		33%-66% of cells	2
		>66% of cells	3
Inflammation	Lobular	No Foci	0
		<2 foci per 200X field	1
		2–4 foci per 200X field	2
		>4 foci per 200X field	3
	Portal	Absence	0
		Presence	1
Ballooning		<5% of cells	0
		5%-33% of cells	1
		>33% of cells	2

### Oil Red O staining and Sudan Black B staining

Livers from subset of wild type (n = 4) and heterozygous (n = 4) mice with macro and/or microvesicular steatosis scores of 0–3 were stained with Oil Red O or Sudan Black B to confirm presence of steatosis. Briefly, cryosectioned livers were washed with running tap water for 10 minutes, rinsed with 60% isopropanol and stained with Oil Red O (Sigma, Cat# O0625-25G) mixed with 60% isopropanol for 15 minutes. The sections were then rinsed with 60% isopropanol, and nuclei were stained with Mayer’s Haematoxylin, rinsed in tap water and coversliped with aqueous mounting medium. For Sudan Black B staining, cryosectioned livers were washed in tap water for 10 minutes, rinsed in 70% ethanol for 1 minute and stained with Sudan Black B (Sigma, Cat# 199664-25G) diluted in 70% ethanol for 8 minutes. The sections were dipped in 70% ethanol for 2 minutes to remove any extra stain, and the nuclei were stained with 0.1% nuclear fast red. Sections were then washed in tap water for 10 minutes and coversliped with aqueous mounting medium. All samples were imaged using Leica microsystem (model DM6000B) and Leica camera (model DFC 450 C).

### Biochemical analysis

Cardiac puncture was performed to collect blood for biochemical analysis. Briefly, after euthanization, blood was collected in Vacuette potassium-EDTA tubes (VWR, Cat #454428) and centrifuged to isolate plasma. Plasma samples were stored at -20°C until analysis. Levels of cholesterol and triglycerides were measured by routine laboratory techniques at the McGill University Health Center core facility.

### RT-PCR

RNA extraction was performed according to Trizol manufacturer's protocol (Invitrogen, Burlington, Ontario). Total RNA was treated with DNAse (NEB, according to manufacturer's protocol) and used for reverse transcription with the iScript^™^cDNA synthesis kit (Bio-rad, Cat. #170–8890, according to the manufacturer's protocol). qRT-PCR was performed using the ssoAdvanced universal SYBR green supermix (Bio-Rad, cat#172–5270) on a Roche LightCycle 480 PCR machine. qPCR experiments were performed in duplicates to ensure technical replicability. At least 4 animals of each genotype were analyzed for biological replicates. Target genes were normalized with the normalization factor as calculated by geNorm software (v3.4; Ghent university hospital center for medical genetics)[21]. Two to three house-keeping genes including B2M, GAPDH, and SDHA were used for the generation of the normalization factor as previously reported [21]. RT-PCR program included a hot start at 95°C for 5 min, followed by 40 cycles of a denaturation step at 95°C for 10s and an annealing/extension step at 60°C for 30s. Primers used in the present study are listed on S1 Table.

### Western blot analysis

Snap-frozen mice tissues were minced and lysed in RIPA buffer (25 mM Tris∙HCl pH 7.6, 10% glycerol, 420 mM NaCl, 2 mM MgCl_2_, 0.5% NP-40, 0.5% Triton X-100, 1 mM EDTA, protease inhibitor) on ice. Approximately 50mg of liver tissue was sonicated and centrifuged at 13000rpm for 20 minutes at 4°C. Clarified protein lysates were measured according to standard methods using a DC protein assay kit (Bio-Rad, Mississauga, Ontario, Canada). Cells were pelleted and lysed with RIPA buffer. 50μg of protein was resolved on 6% and 12% denaturing SDS-polyacrylamide gels and transferred to PVDF membranes, as previously described [22].

For protein samples resolved on TGX Stain-Free gels (Bio-Rad, Cat# 4568045), the gel was activated by exposure to UV light for 1 min to visualize total proteins. They were then transferred to Low Fluorescence PVDF membrane (Bio-Rad, Cat# 1620260), and a stain-free blot image was acquired to obtain a total protein profile. The total protein profile was used as a loading control to normalize the level of the protein of interest. When indicated, β-actin level was used as a loading control to normalize the level of the protein of interest.

After blocking in 5% milk, all membranes were probed with indicated primary antibodies. Immunoblotted proteins were visualized using horseradish peroxidase-conjugated secondary antibodies (Cell Signaling), and antigen-antibody complexes were detected using the ECL system (ZmTech Scientifique, Montreal, Quebec, Canada).

All western blots were repeated at least twice on each sample, and at least 3 animals of each genotype were analyzed for biological replicates. Images of western blots were taken with Bio-Rad’s ChemiDoc MP System. The bands for total proteins and the Chemi images were digitally analyzed using Image Lab software. Primary antibodies used in this study are listed in S2 Table.

### Statistical analysis

Two-tailed unpaired t-test analysis and Fisher’s exact test were calculated using the Prism Software (http://www.graphpad.com/scientific-software/prism/). Significant p-values are represented as * for <0.05, ** for <0.01, *** for <0.001.

## Results

### TMED2 protein was significantly decreased in livers of newborn *Tmed2*^*99J/+*^ mice

**Fig 1:**
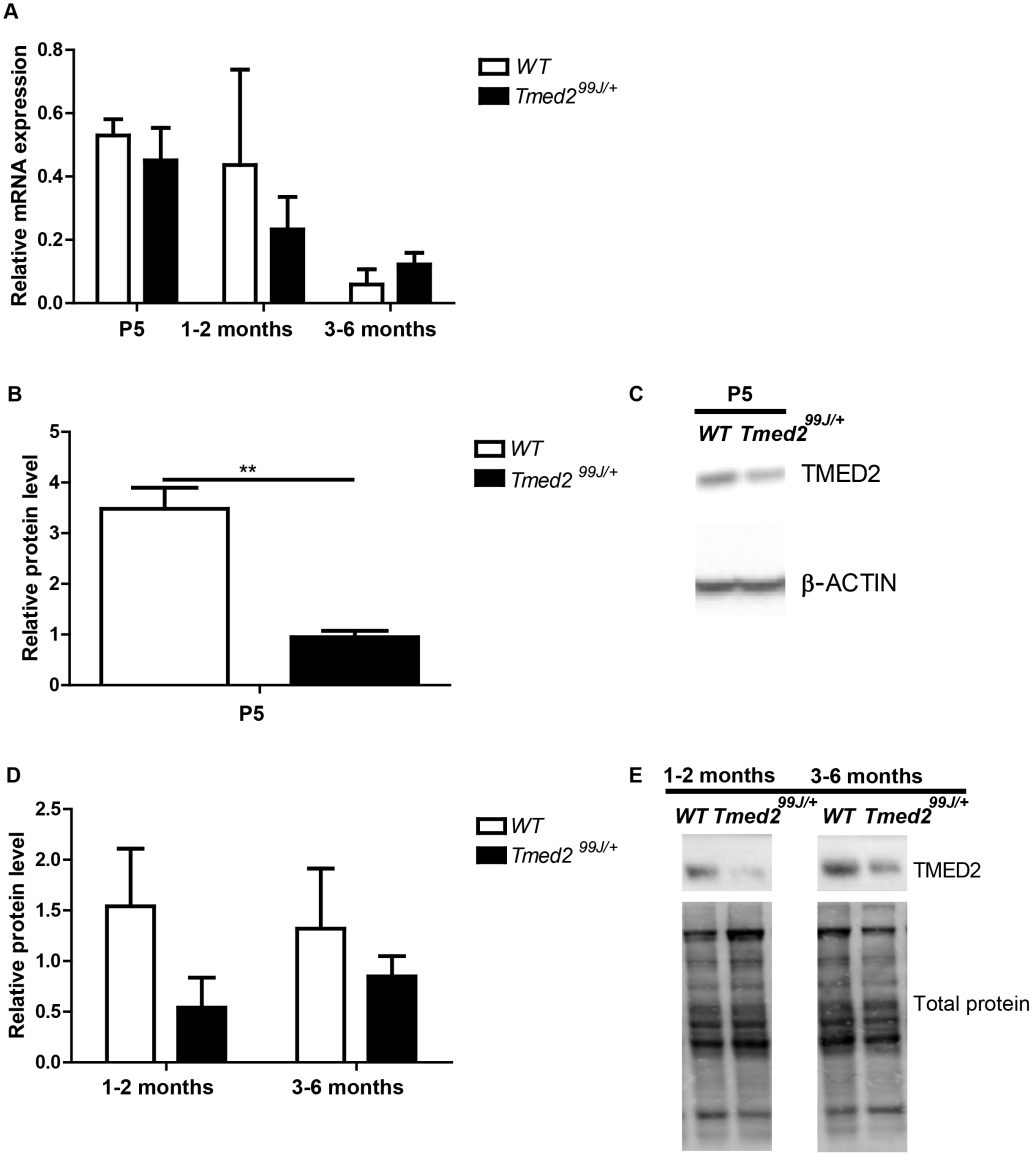
TMED2 level in livers of wildtype and *Tmed2*^*99J/+*^ mice at P5, 1–2 months and 3–6 months. A. RT-qPCR shows no difference in *Tmed2* in livers. B. Western blot analysis showed significantly reduced TMED2 in livers of P5 *Tmed2*^*99J/+*^mice compared to wildtype littermates C. Representative images of Western blot showing expression of TMED2 and β-ACTIN, used as a loading control. D. Reduced TMED2 in livers of 1–2 months and 3–6 months *Tmed2*^*99J/+*^mice compared to age-matched wildtype. E. Representative images of Western blot showing expression of TMED2 and total protein used as loading control. 3 animals of each genotype were analyzed per age group. **P<0.01 by t-test. WT = wildtype.

We previously showed that embryos homozygous mutant for the 99J mutation in *Tmed2* have reduced mRNA and absent protein [5]. To examine the requirement of TMED2 in heterozygous mice, we first quantified *Tmed2* mRNA levels in livers of heterozygous mice carrying the 99J mutation (*Tmed2*^*99/+*^) using qRT-PCR. We predicted that TMED2 levels may correlate with the age and maturity of the mice. Therefore, we analyzed newborn pre-weaning (P5, n = 4 per genotype), post-weaning juvenile (1–2 months, n = 4 per genotype) and adult mice (3 – 6months, n = 4 per genotype), separately. No significant difference was observed in levels of *Tmed2* mRNA in the liver of *Tmed2*^*99/+*^ newborn, juvenile, and adult mice as compared to age-matched wild type mice (Fig 1A). However, TMED2 protein was reduced at all stages analyzed (n = 3 per genotype per age group; Fig 1B–1E), though this decrease was only statistically different when livers of newborn *Tmed2*^*99/+*^mice where compared to age-matched wild type control (two-tailed, unpaired t-test, p = 0.004; Fig 1B and 1C). These data confirmed the previously reported discordance between expression of Tmed2 mRNA and protein, in *Tmed2*^*99/99J*^ embryos [5].

### TMED10 was significantly decreased in livers of newborn *Tmed2*^*99J/+*^ mice

**Fig 2:**
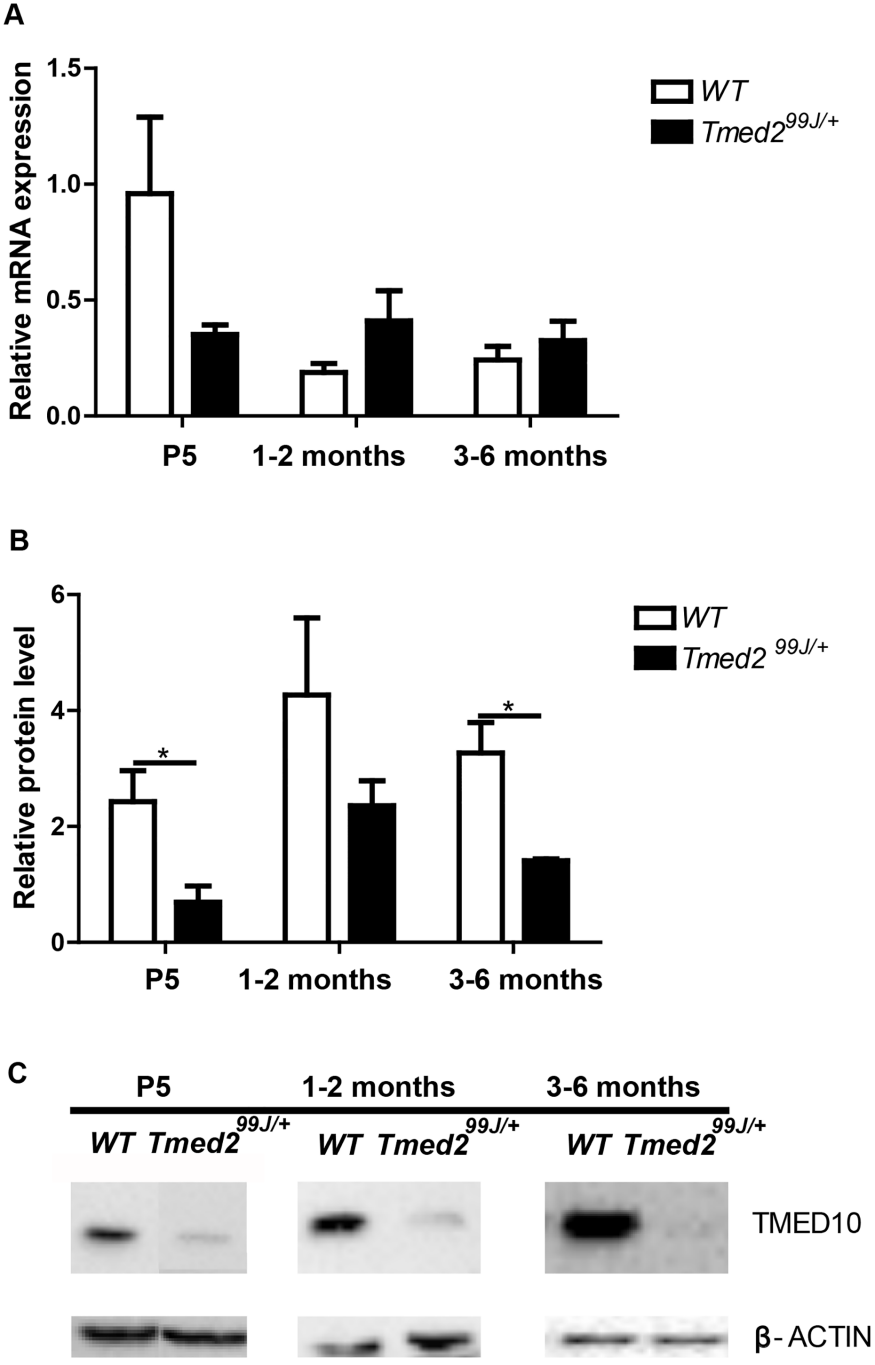
TMED10 level in livers of wildtype and *Tmed2*^*99J/+*^ mice at P5, 1–2 months and 3–6 months. A. RT-qPCR shows no difference in *Tmed2* in livers. B. Western blot analysis revealed significantly decreased TMED10 in livers of *Tmed2*^*99J/+*^mice compared to wildtype littermates at P5 and 3-6months. C. Representative images of Western blot gel showing expression of TMED10 and β-ACTIN, used as a loading control. *P<0.05 by t-test. WT = wildtype.

TMED2 complexes with TMED10 [2, 23, 24], and is required for TMED10 stability and localization[5, 25]}. We examined *Tmed10* mRNA level and found that it was not significantly different between wild type and *Tmed2*^*99/+*^ mice (n = 4 per genotype per age group; Fig 2A). However, TMED10 was reduced at all stages analyzed (n = 3 per genotype per age group; Fig 2B and 2C), and this difference was statistically significant in newborn and adult mice (two-tailed, unpaired t-test, p = 0.045 for newborn, and 0.024 for adult; Fig 2B and 2C). Thus, levels of both TMED2 and its associated partner, TMED10 are decreased in livers of *Tmed2*^*99J/+*^ mice.

### Normal level of TMED2 was not required for tunicamycin induced UPR

Since TMED2 and TMED10 were implicated in rapid ER stress-induced export (RESET), an early step in the unfolded protein response (UPR) important for the degradation of misfolded GPI-anchored proteins [26], we investigated a potential role for TMED2 in the UPR. Treating HepG2 and SKHep1—human liver cancer cell lines—with tunicamycin for 24 hours resulted in a significant increase in glucose regulated protein (GRP)78, consistent with activation of UPR (two-tailed, unpaired t-test, p = 0.048 for HepG2 and 0.029 for SKHep1). However, no significant modulation of TMED2 was found when HepG2 and SKHep1 cells were treated with tunicamycin for 24-hours (S1A–S1D Fig). This indicates that TMED2 was not regulated during tunicamycin-induced UPR. In addition, wild type and *Tmed2*^*99J/+*^ mice treated with tunicamycin, as described previously [27] and in the materials and methods, showed a similar reduction in weight and subsequent recovery (S1E Fig). Overall, our data indicate that TMED2 is not modulated by tunicamycin and suggest that normal levels of TMED2 is not required for tunicamycin-induced UPR.

### ER dilation and increased phosphorylated eIF2α in *Tmed2*^*99J/+*^ livers

**Fig 3:**
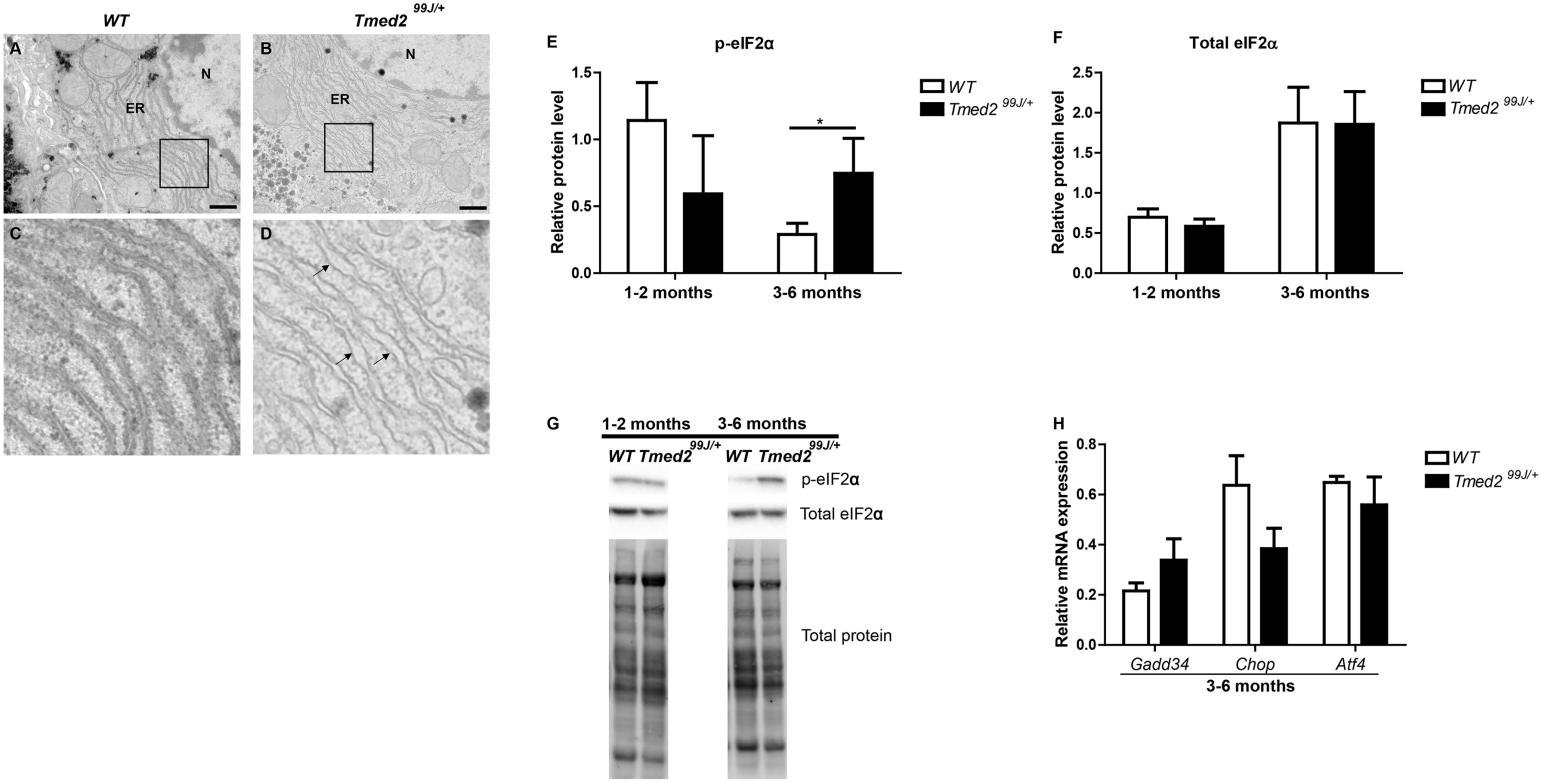
*Tmed2*^*99J/+*^ livers exhibit dilated ER and increased level of the UPR marker phosphorylated-eIF2α. A-D. Transmission Electron Microscopy (TEM) pictures showing dilated ER (arrows) in hepatocytes of *Tmed2*^*99J/+*^ mice (B, D) when compared to wildtype littermates (A, C; scale bar = 500nm). E. Phosphorylated eIF2α (p-eIF2α) was significantly increased in livers of 3–6 months *Tmed2*^*99J/+*^ mice when compared to age-matched wildtype controls. F. No significant difference was found in levels of total eIF2α when *Tmed2*^*99J/+*^ mice were compared to age-matched wildtype controls. G. Representative images of Western blot gel showing expression of p-eIF2α, total eIF2α, and total protein loading control. H. RT-qPCR indicate no significant difference in levels of *Gadd34*, *Chop*, and *Atf4* in livers of 3–6 months *Tmed2*^*99J/+*^ mice when compared to age-matched wildtype controls. 3 animals of each genotype were analyzed per age group, *P<0.05 by t-test. N = nucleus, ER = Endoplasmic Reticulum, WT = Wildtype. Arrows indicate dilated ER.

Golgi of mice with heterozygous mutation in *Tmed10* are moderately dilated [4] therefore, we used transmission electron microscopy to examine the morphology of Golgi and ER in livers of *Tmed2*^*99/+*^ mice. No morphological abnormalities were found in Golgi of *Tmed2*^*99J/+*^ mice (data not shown). However, ER membranes in a subset of adult *Tmed2*^*99J/+*^ mice were mildly dilated (n = 3/4) (Fig 3B and 3D) when compared to age-matched wild type control (Fig 3A and 3C). In one case, severely dilated ER membranes was observed in a hepatocellular carcinoma found in one *Tmed2*^*99J/+*^ mouse (data not shown).

Dilated ER membranes are an indication of ER-stress and are associated with activation of the UPR. Therefore, we examined expression of genes and proteins associated with the UPR [8]. During UPR, expression of chaperone proteins such as GRP78 and GRP94 are increased to reduce the load of unfolded proteins in the ER. In addition, activation of three canonical pathways: eukaryotic translation initiation factor (eIF)-2α Kinase 3 (PERK), endoplasmic reticulum to nucleus signaling/inositol-requiring enzyme (IRE)-1, and activating transcription (ATF)-6, result in transcriptional regulation of genes which will help to maintain ER homeostasis or to initiate cell death [8, 28]. ER-stress was examined in juvenile and/or adult wild type and *Tmed2*^*99J/+*^ mice. No significant differences were found in levels of the chaperone proteins GRP78 and GRP94 (S2A and S2B Fig) in juvenile and adult animals. In addition, splicing of the transcription factor *Xbp1* (S2E Fig)—a surrogate for activation of the IRE-1, and accumulation of cleaved ATF-6 were comparable in livers of adult wildtype and *Tmed2*^*99J/+*^ mice (S2C and S2D Fig). However, phosphorylated eIF2α (peIF2α) was significantly increased in livers of adult *Tmed2*^*99J/+*^ mice, when compared to age-matched wild type mice (two-tailed, unpaired t-test, p = 0.032; Fig 3E–3G), indicating that the PERK arm of the UPR was activated. Nonetheless, expression of *Atf4* and *Chop*, downstream targets of eIF2α were comparable between adult *Tmed2*^*99J/+*^ and wild type mice (Fig 3H). Overall, our data indicate that dilation of ER membranes in *Tmed2*^*99J/+*^ mice was associated with activation of the PERK arm of the UPR.

### Heterozygous *Tmed2* mice develops NAFLD

**Fig 4:**
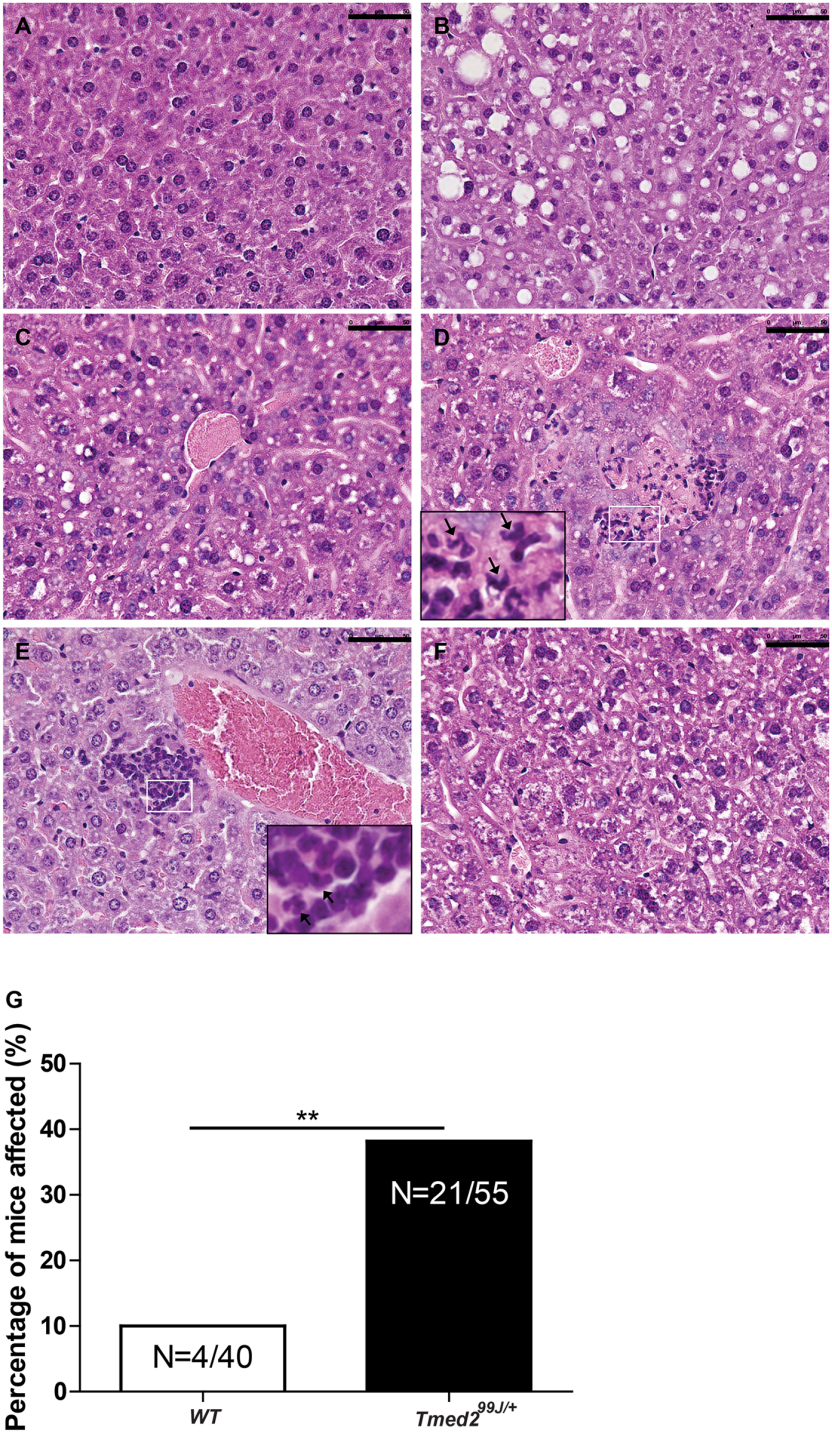
Increased NAFLD in *Tmed2*^*99J/+*^ mice. Representative images of Hematoxylin & Eosin stained liver sections showing A). a healthy liver section; and phenotypes scored for on Table 1; B). macrosteatosis; C). microsteatosis D). lobular inflammation; E). portal inflammation; and F). ballooning. G. Significantly more *Tmed2*^*99J/+*^mice had NAFLD scores of ≥ 4 when compared to age-matched wildtype controls. **P<0.01 using Fisher exact t-test. Arrows indicate inflammatory cells. Scale bar = 50um. WT = Wildtype.

**Fig 5:**
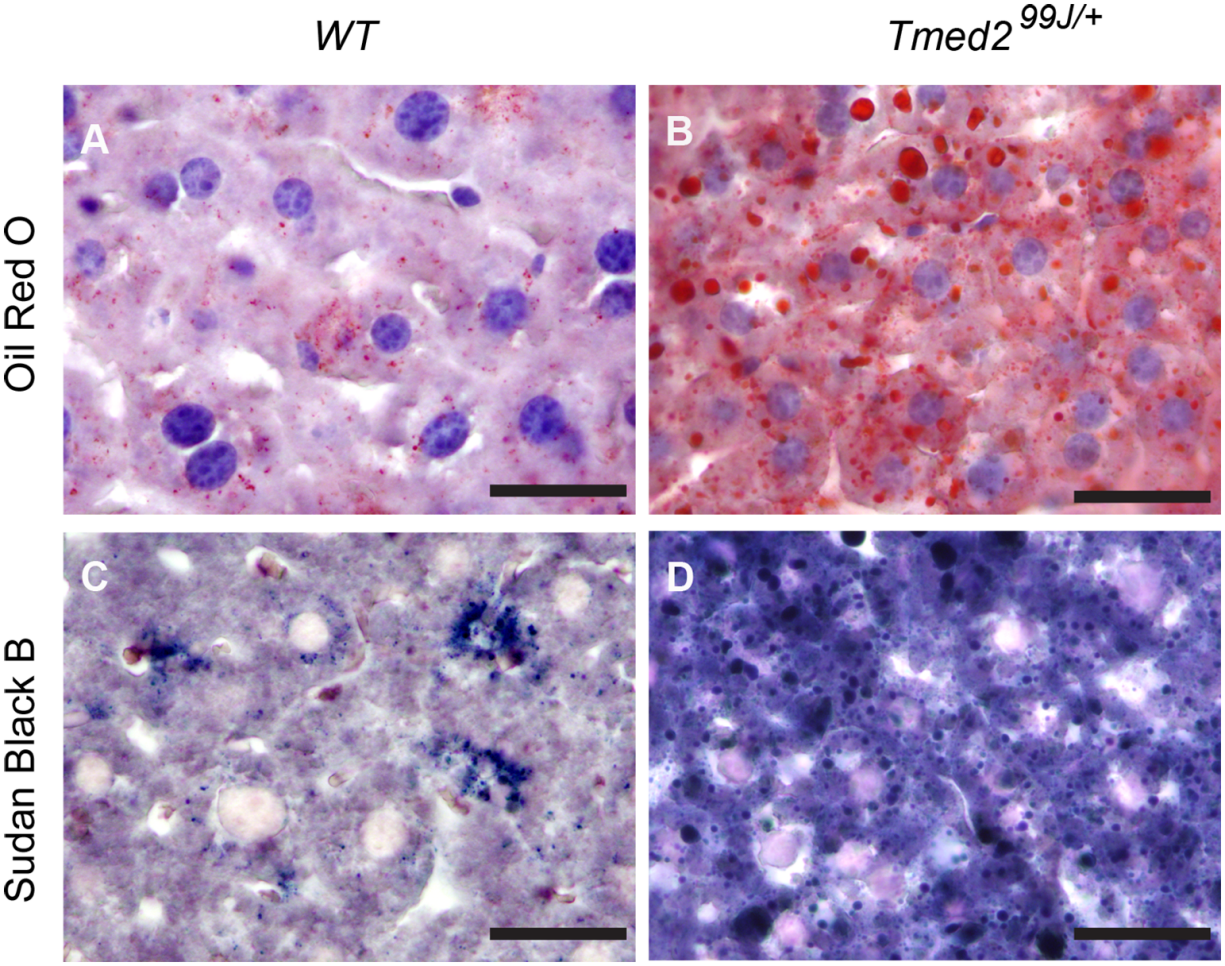
Representative images of liver samples stained with Oil Red O and Sudan Black B. A). wildtype liver with score of 1 had no Oil Red O staining. B). *Tmed2*^*99J/+*^ liver with score of 5 had intense Oil Red O staining. C). Same wildtype sample as in A had no Sudan Black B staining. D). Same *Tmed2*^*99J/+*^ sample as in B had intense Sudan Black B staining. Scale bar = 50um. WT = Wildtype, n = 4 for each genotype.

During our studies, we noted that livers of *Tmed2*^*99J/+*^ mice appeared abnormal and that a subset of mice developed hepatocellular carcinoma, in addition lung and stomach tumors were found in two different *Tmed2*^*99J/+*^mice (manuscript in preparation). To systematically analyze livers of *Tmed2*^*99J/+*^ mice and compare them to their wild type littermates we utilized a modified version of the Non-alcoholic Activity Score (NAS) described by the Non-alcoholic Steatohepatitis Clinical Research Network [20]. Livers from wild type and heterozygous mice between 1 month– 17 months were scored for ballooning, macrosteaosis, and lobular inflammation after Haematoxylin and Eosin (H&E) staining (Table 1). In the course of our analysis, it became apparent that two additional phenotypes, microvesicular steatosis and portal inflammation (Fig 4)- associated with NAFLD [29] and not included in the NAS scoring system, -were also present in wild type and *Tmed2*^*99J/+*^ mice. Hence, these two additional phenotypes were also incorporated into our scoring system (Table 1 and Fig 4). The average age of mice analyzed was not significantly different, 8.6 months and 8.4 months for *Tmed2*^*99J/+*^ and wild type mice, respectively. However, significantly more *Tmed2*^*99J/+*^ mice exhibited NAS activity scores of 4 or higher (n = 21/55) when compared to wild type (n = 4/40) mice (two-tailed, fisher’s exact test, p = 0.0021; Fig 4G). Accumulation of neutral lipids in animals with macrosteatosis score of ≥2 was confirmed after staining with Oil Red O and Sudan Black B (Fig 5). Although, 72–91% of male C3H/HeJ mice were reported to develop hepatomas at the age of 14 months (The Jackson Laboratory), increased susceptibility to liver cancer was not associated with liver disease [30]. Nonetheless, our data indicates that reduction of TMED2 resulted in a 28% increase (from 10% to 38%) in the number of mice with clinical features associated with NAFLD by the age of 6 months.

NAFLD is often associated with a metabolic syndrome [31]. Therefore, features associated with a metabolic syndrome such as increased weight, increased plasma cholesterol and triglycerides were measured in post-weaning juvenile and adult wild type and *Tmed2*^*99J/+*^ mice. No significant difference was found in any of these metabolic indicators when *Tmed2*^*99J/+*^ mice were compared to their wild type littermates (S3 and S4 Figs), although NAFLD was only scored in a subset of mice used for cholesterol and triglyceride studies (n = 5/10 wild type and 3/10 *Tmed2*^*99J*/+^ mice) none of the animals showed any overt liver diseases. However, we noted that plasma triglyceride level was higher and that plasma cholesterol level was lower in adult *Tmed2*^*99J/+*^ mice when compared to age-matched wild type litter mates (S4 Fig). Thus, our data indicates that *Tmed2*^*99J/+*^ mice do not exhibit signs of a metabolic syndrome.

### Levels of *Srebp*1a and *Srebp*2 are increased in *Tmed2*^*99J/+*^ mice

**Fig 6:**
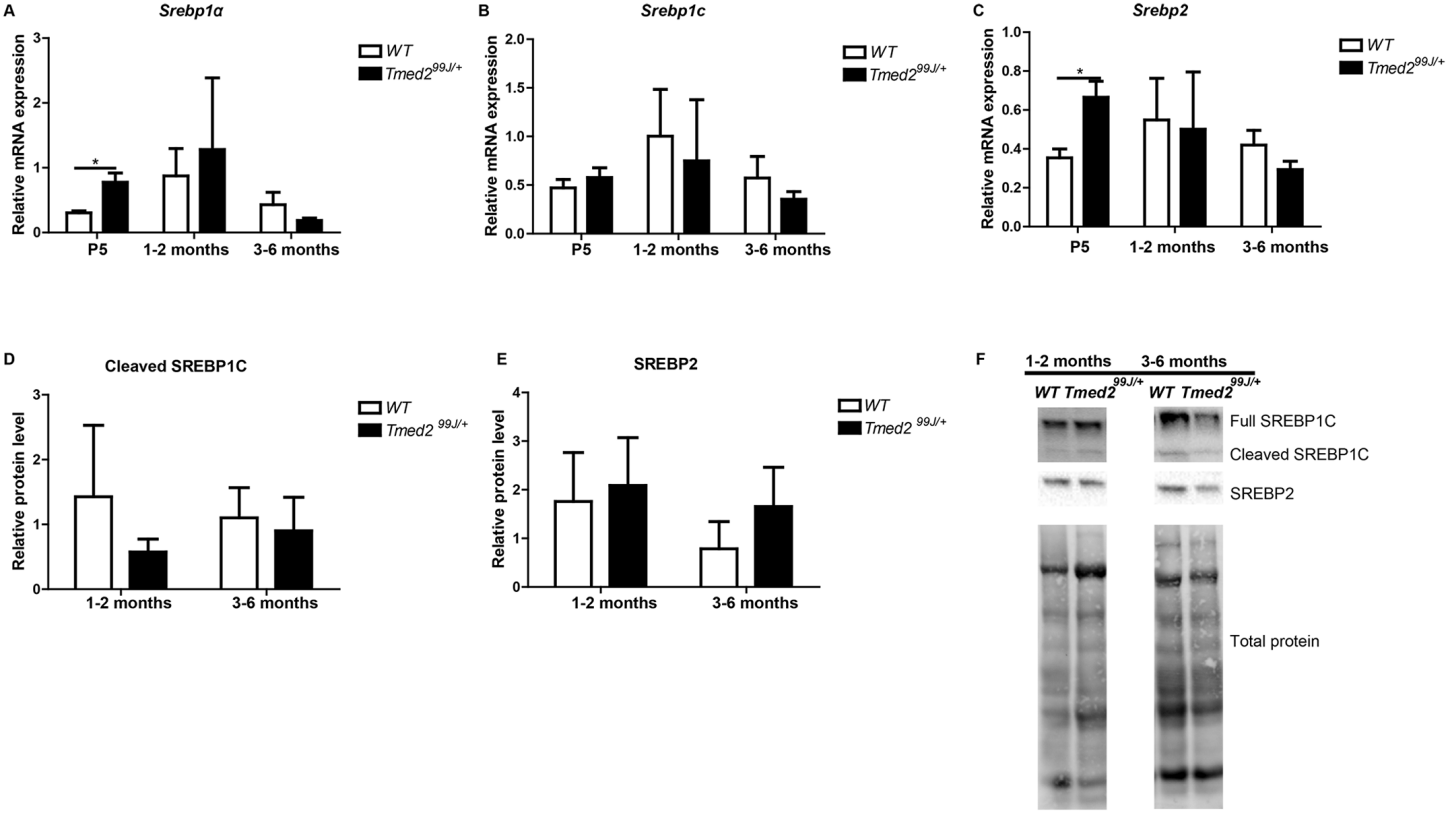
Expression of lipid biosynthesis regulators-SREBPs in wildtype and *Tmed2*^*99J/+*^ livers. A). *Srebp1α* level was increased in *Tmed2*^*99J/+*^ increased in P5 *Tmed2*^*99J/+*^ mice compared to age-matched wildtype controls. B). *Srebp1c* level was comparable in *Tmed2*^*99J/+*^ and age-matched wildtype control mice at all stages. C). *Srebp2* level was increased in P5 *Tmed2*^*99J/+*^ mice compared to age-matched wildtype controls. D). Levels of activated SREBP1C was comparable in *Tmed2*^*99J/+*^ and age-matched wildtype control at 1–2 and 3–6 months. E). Levels of activated SREBP2 was comparable in *Tmed2*^*99J/+*^ and age-matched wildtype control at 1–2 and 3–6 months. F). Representative images of Western blot showing expression of full SREBP1C, cleaved SREBP1C (active form), SREBP2, and total protein loading control. 3 animals of each genotype were analyzed per age group. WT = wildtype. *P<0.05 by t-test.

*Srebp1f* and *Srebp2* belong to a family of transcription factors that regulates fatty acid and cholesterol synthesis, respectively [32–35]. The *Srebp1f* gene encodes for two alternative transcripts: *Srebp1c*, which primarily regulates expression of genes involved in fatty acid biosynthesis, and *Srebp1a* that regulates expression of genes implicated in both fatty acid and cholesterol biogenesis [34]. No significant difference was found in *Srebp1c* expression or in generation of cleaved SREBP1C, when juvenile and adult *Tmed2*^*99J/+*^ mice were compared to wild type age-matched controls (Fig 6B and 6D). Similarly, SREBP2 was comparable between juvenile and adult wild type and *Tmed2*^*99J/+*^ mice (Fig 6E). However, levels of *Srebp1a* and *Srebp2* were significantly higher in livers of newborn *Tmed2*^*99J/+*^ mice when compared to age-matched wild type littermates (two-tailed, unpaired t-test, p = 0.017 for *Srebp1a*, and 0.016 for *Srebp2*; Fig 6A and 6C). Thus, expression of transcription factors associated with steatosis and NAFLD is increased in newborn *Tmed2*^*99J/+*^ mice.

## Discussion

### A novel model of NAFLD

Herein, we describe a novel model of NAFLD associated with haploinsufficiency for the cargo receptor TMED2. We showed that newborn mice carrying the 99J point mutation in *Tmed2* have a significant decrease in TMED2 and TMED10 with no associated change in the mRNAs. In addition, TMED2 was not regulated by the UPR and normal levels of TMED2 was not required for tunicamycin-associated UPR. However, livers from *Tmed2*^*99/+*^ mice had dilated ER membranes and activation of the PERK arm of the UPR pathway, as indicated by increased phosphorylation of eIF2α [36]. Consistent with the established association of the UPR in liver disease, *Tmed2*^*99/+*^ mice develop NAFLD with no associated metabolic disease [8]. Increased expression of *Srebp2* and *Srebp1a* in livers of newborn heterozygous mice is postulated also to contribute to the development of NAFLD in adult *Tmed2*^*99J/+*^ mice.

### TMED2 regulates stability of TMED10

Expression levels of TMED proteins are interdependent [2–5], and reduction of TMED2 was associated with a reduction of TMED10 in *Tmed2*^*99J/+*^ mice. TMED2 and TMED10 form hetero-oligomeric complexes in the early secretory pathway [2] and where TMED10 is predicted to aid in retrieval of TMED2 and other ER-resident proteins from the Golgi in TMED10-associated COPI-coated vesicles [2, 3, 37]. In addition, although interactions between TMED proteins are essential for individual TMED protein stability, interacting-TMED proteins are not always expressed or required in the same cells and organelles [5, 9–11]. In fact, Jenne et al showed that TMED protein oligomeric complexes are organelle specific, [2]and Strating et al proposed that through an unknown mechanism individual TMED proteins provide proper ER/Golgi sub-compartmental environment during transport [11]. Our studies and those of Denzel et al showing that TMED2 and TMED10 are required for normal ER and Golgi morphology, respectively [4, 11] are consistent with the hypothesis proposed by Strating et al [11]. Current studies in our laboratory aim to identify the TMED2 specific partners important for its dependent and independent functions in the liver.

### Normal levels of TMED2 is required for normal ER-homeostasis but not for UPR

In the presence of ER-stress, GRP78 dissociates from three ER-transmembrane resident proteins—PERK, IRE1α, and ATF6—to initiate a cascade of activity that either resolves the stress or promotes cell death [8]. PERK phosphorylates eIF2α, which attenuates general protein translation to reduce the influx of nascent and unfolded polypeptide chains into the ER [38]. In parallel, eIF2α phosphorylation increases the translation of a subset of mRNAs, for example, *Atf4* to activate expression of pro-apoptotic genes such as C/EBP homologous protein (*Chop*) and growth arrest and DNA damage-inducible protein (*Gadd34*) [39]. Activation of IRE1α results in alternative splicing of the mRNA encoding for X-box binding protein 1 (*Xbp1*) that is involved in regulation of molecular chaperones such as *Grp78* and *Grp94* [40]. On the other hand, ATF6 translocates to the Golgi where it is proteolytically cleaved and released to the nucleus to activate transcription of target genes including ER chaperones and ER-associated protein degradation (ERAD) components [41].

As cargo receptors are important for transport of proteins from the ER, it was expected that reduction or mutations in TMED proteins would lead to UPR, or be required to mount an effective UPR. In fact, deletion of emp24, the yeast ortholog of *Tmed2*, results in splicing of *Xbp1* and secretion of bip/Grp78, two markers of UPR [6]. However, in drosophila, mutations in Tmed genes result in activation of the NF-κB pathway and expression of genes consistent with activation of the PERK arm of the UPR. Intriguingly, no evidence of *Xbp1* alternative splicing was found suggesting that a specific arm of the UPR is activated in Tmed mutants [7]. Our group previously reported that homozygous loss of function mutations in *Tmed2* did not result in *Xbp1* alternative splicing, indicating that the IRE-1α arm of the UPR was not activated [5]. In the current study, we confirmed that the IRE-1α and the ATF-6 arms of the UPR were not activated. However, we found that TMED2 was required for normal ER-morphology and that reduced levels of TMED2 was associated with activation of the PERK arm of the UPR. Considering the observations of Bolz and Carney [7]and our findings, we propose that the activation of the PERK arm of the UPR is a conserved mechanism through which animal cells responds to perturbation of TMED protein levels. In addition, we found that expression of genes associated with activation of the PERK arm of the UPR, such as *Atf4*, *Chop*, and *GADD34* was not increased in these mice, as was reported in a subset of patients with NAFLD [36]. Future work in our model will focus on determining if the NF-κB pathway is also activated and potentially contributing to development of NAFLD in *Tmed2*^*99/+*^ mice. We will also investigate if failure to activate expression of genes downstream of the PERK arm of the UPR contributes to NAFLD in *Tmed2*^*99/+*^ mice.

Satpute-Krishan et al., showed a requirement for TMED10 in export of misfolded GPI- anchored proteins downstream of thapsigargin or Dithiothreitol (DTT)-mediated ER-stress, prior to UPR [26]. Since TMED2 and TMED10 interact, and levels of TMED10 were decreased in *Tmed2*^*99/+*^ mice, we reasoned that TMED2 may be regulated by the UPR or like TMED10 be required for UPR. However, we found no evidence to support a similar requirement for TMED2 in UPR. Though unlikely, it is possible that the type of ER-stress may dictate if TMED proteins are required. Thus, thapsigargin induced ER-stress—which disrupts calcium flux, and DTT induced ER-stress—which blocks disulfide bond formation in polypeptides, may be more dependent on the amount of TMED cargo receptors than tunicamycin induced ER stress, which blocks N-glycosylation and glycoprotein biosynthesis at the first step. Alternatively, RESET could be specifically dependent on TMED10 and not TMED2. Treating TMED10 heterozygous mice with ER-stress inducing reagents will help to resolve this discrepancy.

### PERK activation and increased expression of *Srebp2* and *Srebp1a* in *Tmed2*^*99/+*^ mice

Increased levels of *Srebp2* and *Srebp1a* in newborn *Tmed2*^*99J/+*^ mice suggest that signals downstream of TMED2 regulate cholesterol and triglyceride metabolism in newborn mice. However, although we were unable to determine if increased *Srebp2* and *Srebp1a* was associated with an increase in active protein; levels of *Srebp1a* and *Srebp2* mRNA and proteins were comparable in juvenile and adult *Tmed2*^*99J/+*^ mice, when compared to age-matched wild type mice, and hypercholestoremia and hypetriglyceridemia were not observed in *Tmed2*^*99J/+*^ mice. These findings indicate that continued dysregulation of SREBP1 and SREBP2 was not responsible for NAFLD in this mutant mouse line. In contrast, a direct role for the PERK-peIf2α -ATF4 pathway has been established in steatosis in mouse models and corroborated in human. Thus, we propose that phosphorylated eIF2α partly contributes to NAFLD in *Tmed2*^*99J/+*^ mice. We hypothesize that reduced TMED2 leads to abnormal ER homeostasis and UPR. Constitutive activation of UPR or an inability to trigger downstream events in the UPR in *Tmed2*^*99J/+*^ mice in turn increase susceptibility of these mice to unknown factors that promote NAFLD in a subset of *Tmed2*^*99J/+*^ mice. Patients with NAFLD can be grouped base on four different phenotypes: obese, type 2 diabetes, metabolic syndrome, and lean patients [42].We expect that this mouse model will be instrumental in future studies aimed to identify the genetic and cellular factors involved in non-obese or lean NAFLD patients [43].

### TMED proteins in diseases

Though discovered over twenty years ago, the requirement and function of the TMED family has long remained an enigma. However, recently, the contribution of TMED family members in diseases has begun to emerge. Disrupted expression of TMED proteins is associated with a diverse group of diseases ranging from cancer to Alzheimer’s. TMED3 is an emerging tumor suppressor gene implicated in prostate cancer [44], colon cancer [45] and hepatocellular carcinoma progression [46]. Intriguingly, in The Exome Aggregation Consortium no loss of function mutations have been reported in TMED2 and half the expected number of missense mutations have been found, suggesting that this gene is essential in human. Our work presented here indicates that TMED2 is a candidate gene in a human disease, specifically NAFLD.

## Supporting information

S1 Fig TMED2 expression is not regulated by Tunicamycin or required for Tunicamycin-induced stress. A). Tunicamycin induces increased level of the unfolded protein response marker, GRP78 in HepG2 cells when compared to vehicle-treated controls. The same treatment did not affect TMED2. B). Representative images of Western blot gel showing expression of TMED2, GRP78 and β-actin loading control. C). Tunicamycin induces increased level of the unfolded protein response marker, GRP78 in tunicamycin treated SKHep1 cells when compared to vehicle-treated controls. The same treatment did not affect TMED2. D). Representative images of Western blot gel showing expression of TMED2, GRP78 and β-actin loading control. E). Percent weight loss in wildtype and *Tmed2*^*99J/+*^ mice after tunicamycin injection at age of 10 weeks. n = 4 for wildtype and n = 6 for *Tmed2*^*99J/+*^ mice. WT = wildtype, Veh = Vehicle, Tuni = Tunicamycin.
(TIF)

S2 Fig Expression of genes associated with the unfolded protein response (UPR) are not disrupted in *Tmed2*^*99J/+*^ mice. A). GRP78 level was comparable in livers of *Tmed2*^*99J/+*^ and stage-matched wildtype controls. B). level of GRP94 was comparable in livers of *Tmed2*^*99J/+*^ and stage-matched wildtype controls. C). Level of activated ATF6α was comparable in livers of 3–6 months *Tmed2*^*99J/+*^ and stage-matched wildtype controls. D. Representative images of Western blot gel showing expression of GRP78, GRP94, cleaved ATF6α, total ATF6α and total protein internal controls. E.) Levels of spliced *Xbp1* and unspliced *Xbp1* were comparable in livers of 3–6 months wildtype and *Tmed2*^*99J/+*^ mice. n = 3 for each genotype. WT = wildtype.
(TIF)

S3 Fig No significant differences in body and liver weight of *Tmed2*^*99J/+*^ mice when compared to age-matched controls. A). Bar graph showing body weight of *Tmed2*^*99J/+*^ and age-matched wildtype controls. B). Bar graph showing liver weight of *Tmed2*^*99J/+*^ and age-matched wildtype controls. C). Bar graph showing percentage of liver to body weight ratio in both wildtype and *Tmed2*^*99J/+*^ mice. n = 3 for wildtype and n = 4 for *Tmed2*^*99J/+*^ mice for 1–2 months age group; n = 11 for wildtype and n = 10 for *Tmed2*^*99J/+*^ mice for 3–6 months age group. WT = wildtype.
(TIF)

S4 Fig No significant difference in circulating cholesterol and triglycerides levels in wildtype and *Tmed2*^*99J/+*^ mice. A). Plasma cholesterol levels were comparable between wildtype and *Tmed2*^*99J/+*^ at 1–2 months, but B). decreased in *Tmed2*^*99J/+*^ mice at 3–6 months age-matched wildtype controls (P = 0.07, t-test). Plasma triglycerides levels were comparable between wildtype and *Tmed2*^*99J/+*^ at 1–2 months but D). increased in *Tmed2*^*99J/+*^ mice at 3–6 months when compared to age-matched wildtype controls (P = 0.06, t-test). n = 5 for wildtype and n = 4 for *Tmed2*^*99J/+*^ mice for 1–2 months age group; n = 5 for wildtype and n = 6 for *Tmed2*^*99J/+*^ mice for 3–6 months age group. WT = wildtype.
(TIF)

S1 Table List of primers used in RT-qPCR analysis. (DOCX)

S2 Table List of antibodies used in Western blot analysis. (DOCX)
