# Supplementary figures and images for: Non-alcoholic fatty liver disease in mice with heterozygous mutation in TMED2
Source: PLoS One. 2017 Aug 10;12(8):e0182995. doi: 10.1371/journal.pone.0182995 (PMC5552249; doi:10.1371/journal.pone.0182995)

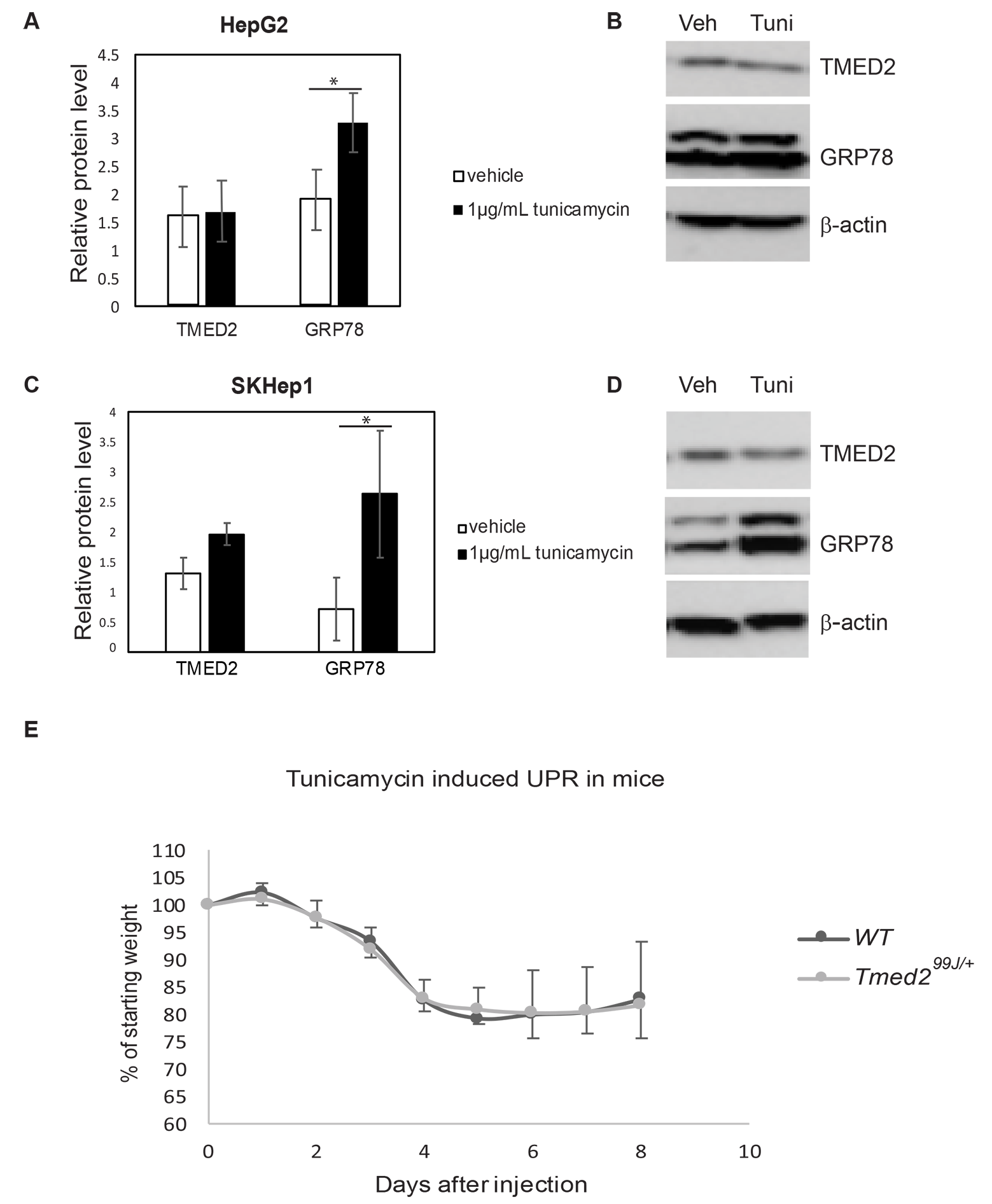

Supplement: S1 Fig — A). Tunicamycin induces increased level of the unfolded protein response marker, GRP78 in HepG2 cells when compared to vehicle-treated controls. The same treatment did not affect TMED2. B). Representative images of Western blot gel showing expression of TMED2, GRP78 and β-actin loading control. C). Tunicamycin induces increased level of the unfolded protein response marker, GRP78 in tunicamycin treated SKHep1 cells when compared to vehicle-treated controls. The same treatment did not affect TMED2. D). Representative images of Western blot gel showing expression of TMED2, GRP78 and β-actin loading control. E). Percent weight loss in wildtype and Tmed299J/+ mice after tunicamycin injection at age of 10 weeks. n = 4 for wildtype and n = 6 for Tmed299J/+ mice. WT = wildtype, Veh = Vehicle, Tuni = Tunicamycin. (TIF) [file pone.0182995.s001.tif]

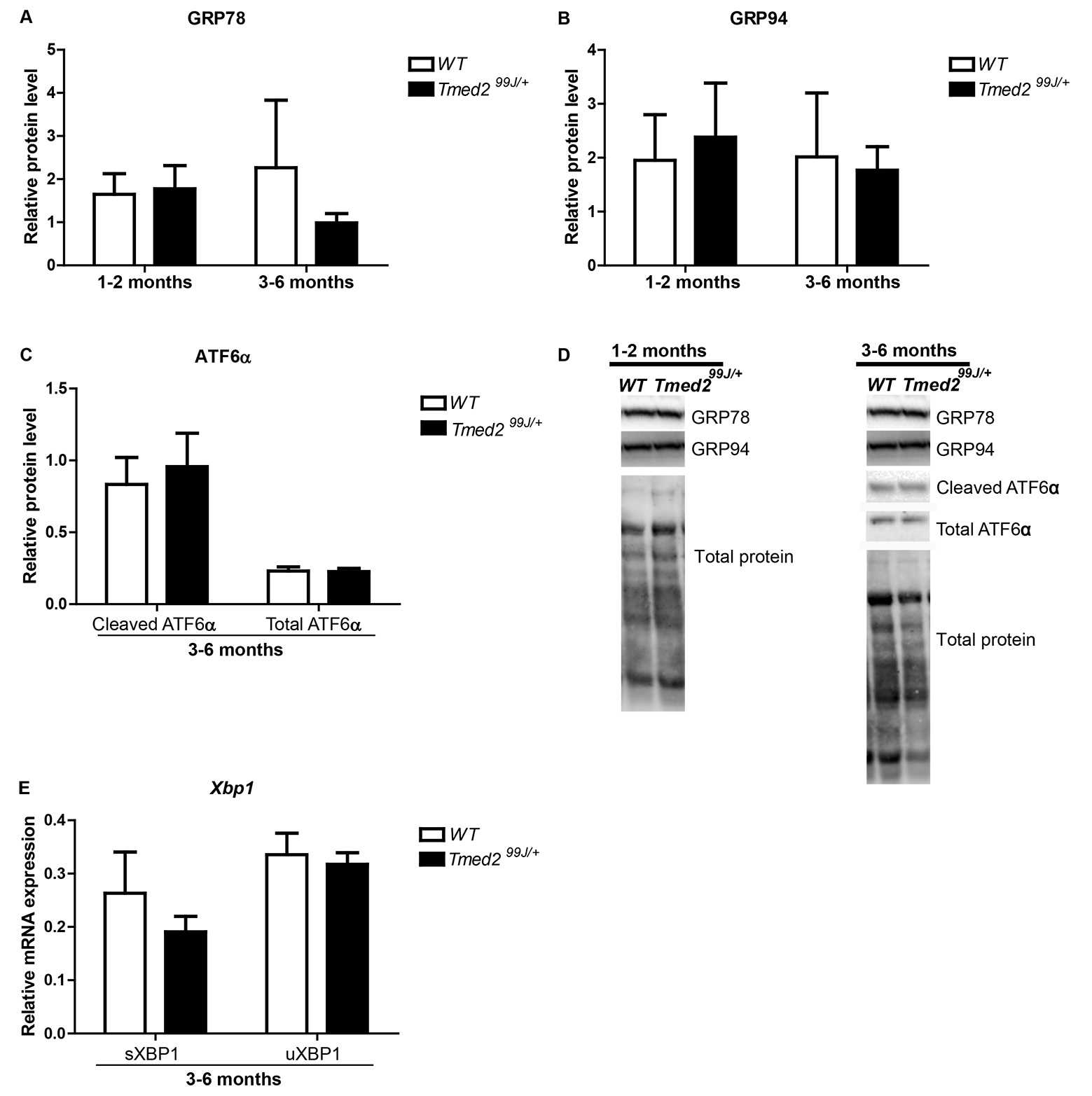

Supplement: S2 Fig — A). GRP78 level was comparable in livers of Tmed299J/+ and stage-matched wildtype controls. B). level of GRP94 was comparable in livers of Tmed299J/+ and stage-matched wildtype controls. C). Level of activated ATF6α was comparable in livers of 3–6 months Tmed299J/+ and stage-matched wildtype controls. D. Representative images of Western blot gel showing expression of GRP78, GRP94, cleaved ATF6α, total ATF6α and total protein internal controls. E.) Levels of spliced Xbp1 and unspliced Xbp1 were comparable in livers of 3–6 months wildtype and Tmed299J/+ mice. n = 3 for each genotype. WT = wildtype. (TIF) [file pone.0182995.s002.tif]

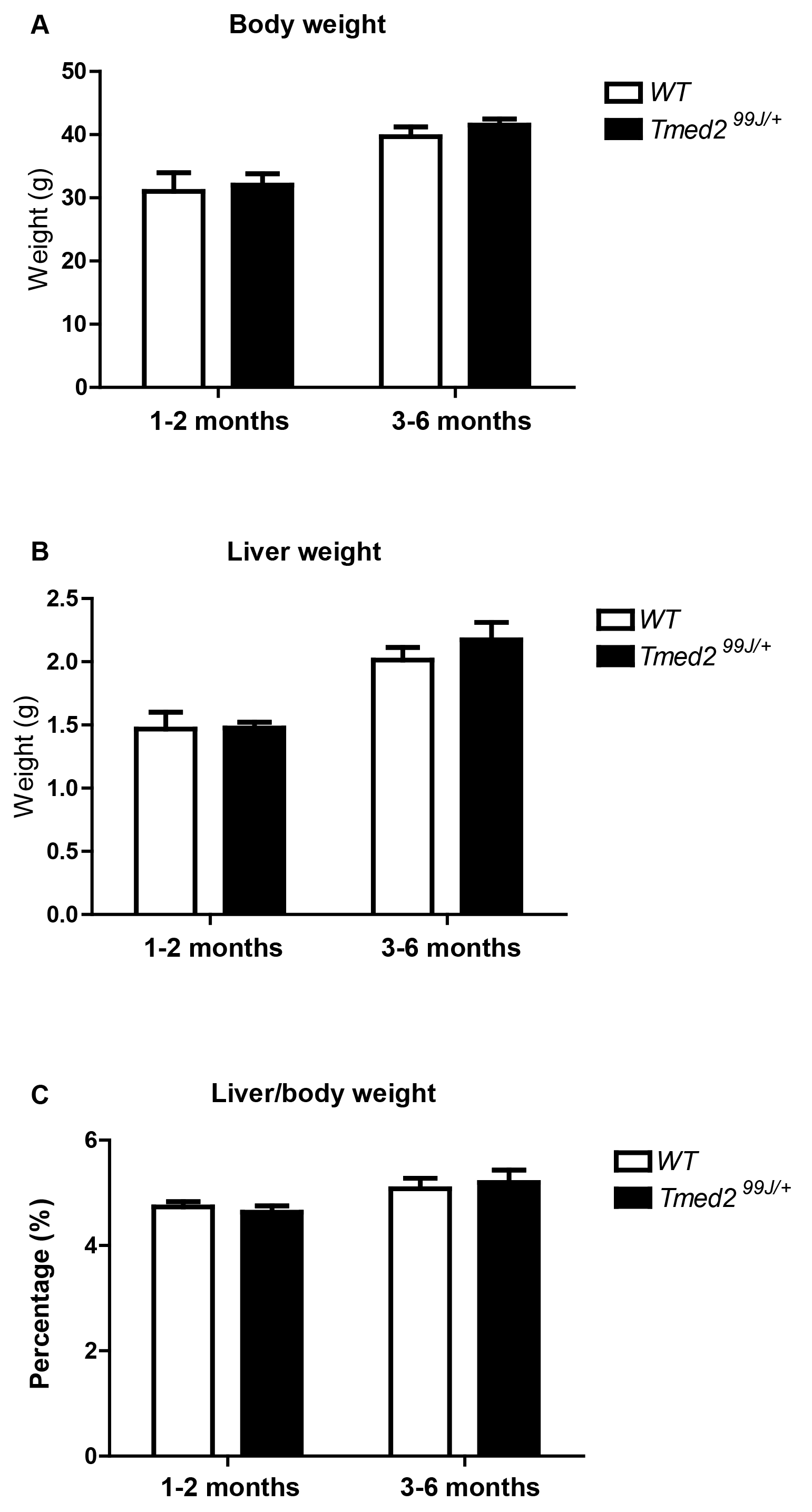

Supplement: S3 Fig — A). Bar graph showing body weight of Tmed299J/+ and age-matched wildtype controls. B). Bar graph showing liver weight of Tmed299J/+ and age-matched wildtype controls. C). Bar graph showing percentage of liver to body weight ratio in both wildtype and Tmed299J/+ mice. n = 3 for wildtype and n = 4 for Tmed299J/+ mice for 1–2 months age group; n = 11 for wildtype and n = 10 for Tmed299J/+ mice for 3–6 months age group. WT = wildtype. (TIF) [file pone.0182995.s003.tif]

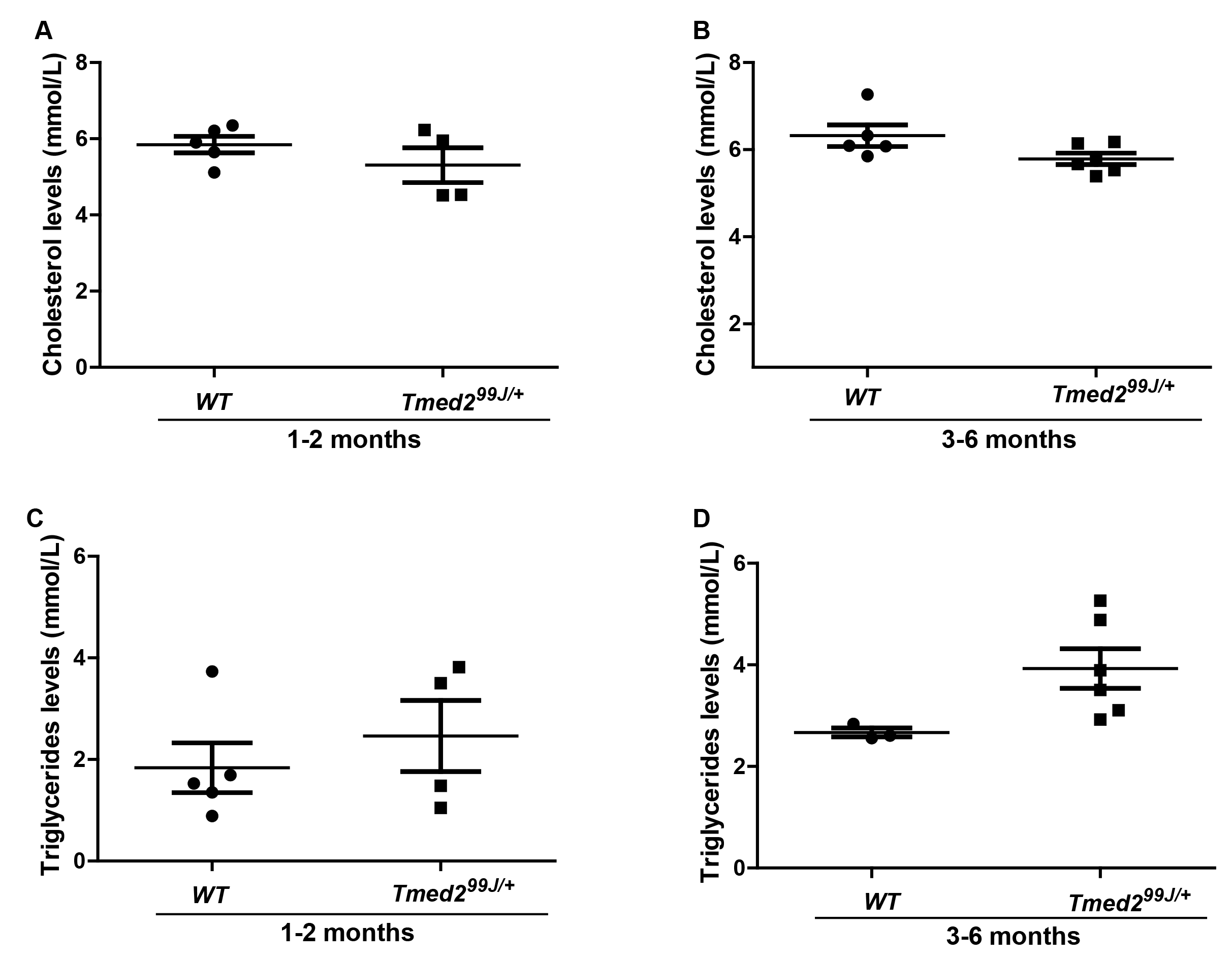

Supplement: S4 Fig — A). Plasma cholesterol levels were comparable between wildtype and Tmed299J/+ at 1–2 months, but B). decreased in Tmed299J/+ mice at 3–6 months age-matched wildtype controls (P = 0.07, t-test). Plasma triglycerides levels were comparable between wildtype and Tmed299J/+ at 1–2 months but D). increased in Tmed299J/+ mice at 3–6 months when compared to age-matched wildtype controls (P = 0.06, t-test). n = 5 for wildtype and n = 4 for Tmed299J/+ mice for 1–2 months age group; n = 5 for wildtype and n = 6 for Tmed299J/+ mice for 3–6 months age group. WT = wildtype. (TIF) [file pone.0182995.s004.tif]
