# Supplementary material for: Non-alcoholic fatty liver disease in mice with heterozygous mutation in TMED2
Source: PLoS One. 2017 Aug 10;12(8):e0182995. doi: 10.1371/journal.pone.0182995 (PMC5552249; doi:10.1371/journal.pone.0182995)
Supplement: S1 Table — (DOCX) [file pone.0182995.s005.docx]

**S1 Table.** Primers used for quantitative real time PCR.

| **Gene** | **GenBank Accession Number^1^** | **Forward Primer** | **Reverse Primer** |
| --- | --- | --- | --- |
| *Gapdh* | NM_001001303.1 | ATGACATCAAGAAGGTCCTG | CATACCAGGAAATGAGCTTG |
| *Sdha* | NM_023281.1 | GCTGTGGCCCTGAGAAAGATC | ATCATGGCCGTCTCTGAAATTC |
| *B2m* | NM_009735.3 | ATGCTATCCAGAAAACCCCTCAA | GCGGGTGGAACTGTGTTACG |
| *Tmed2* | NM_019770.2 | CGGACAACAGGAGTACATGGAAGTCCG | GACCAAAGGACCACTCTGCTGT |
| *Tmed10* | NM_026775.4 | GGAGGTGGAGTTACGACGG | TGGACTCATTAGTGTCCCTCATC |
| *Srebp1c* | NM_001313979.1 | GGAGCCATGGATTGCACATT | GGCCCGGGAAGTCACTGT |
| *Srebp1a* | NM_011480.4 | TAGTCCGAAGCCGGGTGGGCGCCGGCGCCAT | GATGTCGTTCAAAACCGCTGTGTGTCCAGTTC |
| *Srebp2* | NM_033218.1 | GATGAGCTGACTCTCGGGGACATC | GTGGGGTAGGAGAGACTTTGACCT |

1National Center for Biotechnology Information (NCBI)
