# Supplementary material for: Non-alcoholic fatty liver disease in mice with heterozygous mutation in TMED2
Source: PLoS One. 2017 Aug 10;12(8):e0182995. doi: 10.1371/journal.pone.0182995 (PMC5552249; doi:10.1371/journal.pone.0182995)
Supplement: S2 Table — (DOCX) [file pone.0182995.s006.docx]

**S2 Table.** Primary antibodies used for immunoblotting.

| **Antibody** | **Type** | **Host** | **Concentration or dilution** | **Supplier** |
| --- | --- | --- | --- | --- |
| TMED2 | Polyclonal | Rabbit | 1:4000 | Majewska *et al.* |
| TMED10 (TMP21) | Polyclonal | Rabbit | 1:1000 | Cell Signaling |
| GRP78 | Polyclonal | Rabbit | 1:4000 | Abcam |
| GRP94 | Polyclonal | Rabbit | 1:4000 | Abcam |
| eIF-2α | Polyclonal | Rabbit | 1:2000 | Cell Signaling |
| eIF-2α P | Monoclonal | Rabbit | 1:2000 | Epitomics |
| ATF6α | Monoclonal | Mouse | 1:1000 | Novus Biolabs |
| SREBP2 (SREBF2) | Polyclonal | Rabbit | 1:1000 | Protein Tech |
| SREBP1c | Monoclonal | Mouse | 1:1000 | Abcam |
